# Drug Discovery in Liver Disease Using Kinome Profiling

**DOI:** 10.3390/ijms22052623

**Published:** 2021-03-05

**Authors:** Bingting Yu, Ruslan Mamedov, Gwenny M. Fuhler, Maikel P. Peppelenbosch

**Affiliations:** Department of Gastroenterology and Hepatology, Erasmus MC—University Medical Center Rotterdam, 3015 CN Rotterdam, The Netherlands; b.yu@erasmusmc.nl (B.Y.); rmamedov2000@yahoo.com (R.M.); g.fuhler@erasmusmc.nl (G.M.F.)

**Keywords:** kinases, kinome, liver, hepatocytes, hepatitis, hepatocellular carcinoma, peptide arrays

## Abstract

The liver is one of the most important organs, playing critical roles in maintaining biochemical homeostasis. Accordingly, disease of the liver is often debilitating and responsible for untold human misery. As biochemical nexus, with kinases being master regulators of cellular biochemistry, targeting kinase enzymes is an obvious avenue for treating liver disease. Development of such therapy, however, is hampered by the technical difficulty of obtaining comprehensive insight into hepatic kinase activity, a problem further compounded by the often unique aspects of hepatic kinase activities, which makes extrapolations from other systems difficult. This consideration prompted us to review the current state of the art with respect to kinome profiling approaches towards the hepatic kinome. We observe that currently four different approaches are available, all showing significant promise. Hence we postulate that insight into the hepatic kinome will quickly increase, leading to rational kinase-targeted therapy for different liver diseases.

## 1. Introduction

The liver is the largest organ of human body and serves as the nexus of human physiological chemistry. Thus, liver disease is the cause of untold misery [1]. The etiology of liver diseases is manifold, but important causes for pathology are viral infection (e.g., Hepatitis B or C), alcoholic or non-alcoholic steatosis, and oncological transformation, in addition to a large number of smaller etiologies. Although substantial progress has been made, especially with regard to the prevention and treatment of hepatitis, there is still a clear clinical need for improved pharmacological options for the treatment for liver disease (e.g., [2]). In this respect, the superfamily of protein kinases attracts substantial interest. Due to the evolutionary conserved ATP-binding pocket, kinases are highly and often specifically druggable [3] while these enzymes act as the master regulators of cellular biochemistry and thus their inhibition can substantially alter cell behavior [4]. As the liver is biochemically an unusually active organ it is to be expected that targeting kinases should prove exceedingly useful for combating hepatic pathology. Indeed the success of the kinase inhibitor sorafenib in the treatment of otherwise medication resistant hepatocellular carcinoma shows the promise, which targeting the kinase compartment holds in this respect [5]. Unfortunately, kinase action in the liver is substantially different from that observed at other places of the body, hampering design of rational kinase-targeted therapy (e.g., [6]). Kinome profiling technology, which generates comprehensive descriptions of cellular kinase activity without a priori assumptions of the kinase enzymes present in a biological sample represents an obvious way forward in this respect [7]. Here we review the potential of kinase profiling for different forms of liver disease. To this end we shall first shortly emphasize the role of kinases in liver disease and the potential for identifying novel kinase targets therein, followed by an exploration of the potential technical approaches for kinome profiling in clinical liver samples and preclinical model systems of hepatic disease.

## 2. Viral Infection

The most prevalent etiology of hepatitis is viral and viral hepatitis represents an immense burden on human society, e.g., it is estimated that in 2013 viral hepatitis provoked more as 1.5 million fatalities worldwide. Death due to viral infection can be a direct result of acute viral hepatitis, or due to development of cirrhosis or cancer upon development of chronic viral infection [8]. Several hepatitis viruses are recognized, with hepatitis A, B, and C the best known of these. Hepatitis B and C together account for 96% of all hepatitis-related mortality [9]. Hepatitis D and E are as yet less prevalent, although their health burden is increasingly recognized. For instance, the global pooled prevalence for hepatitis D is estimated at 0.8% of the population [10] while approximately 939 million people have ever experienced hepatitis E virus (HEV) infection [11]. In the majority of cases, viral hepatitis can be prevented or treated. Hepatitis A and hepatitis B can be prevented by vaccination [12]. Effective treatments for hepatitis C are available but costly [13]. However, a substantial number of cases viral hepatitis is persistent even in the presence of contemporary treatment options and it is hoped that improved insight into molecular details of disease etiology can offer new hope for the patients involved [14].

Generally speaking, effective immunity against viral infection of hepatocytes depends on the so-called interferon-stimulated genes (ISGs), a group of proteins that is essential for combating pathogens. Transcription of ISGs occurs rapidly upon viral entry into the cytoplasm and this is conventionally associated with the activation of the Janus kinase/signal transducer and activator of the transcription (JAK-STAT) pathway, for instance in response to interferons [15]. However, recent work from our group has recently uncovered a variety of non-canonical mechanisms regulating ISG transcription that open possibilities for improving antiviral immunity through modulation of kinase activities. Interesting observations in this respect are the identification of a role for unphosphorylated STAT1 and unphosphorylated ISG3 in inducing ISG production [16,17], the discovery that certain protein kinase C (PKC) isoforms constrain viral replication in the liver [18], and the role of nuclear factor κ B (NF- κB) in counteracting viral replication in hepatocytes [19,20]. Antiviral actions of interferons are also mediated through the effector Protein Kinase R (PKR) [21]. Additionally, it has been demonstrated that the HBV replication may be reduced by the p70 ribosomal S6 kinase (p70S6K), which blocks the autophagic pathway initiated by AMP-kinase (AMPK) [22]. Conversely, antagonism of spleen tyrosine kinase (SYK) using pharmacological kinase inhibitors was shown to attenuate the progression of viral hepatitis [23]. Viral infection itself also activates the p38 mitogen activated kinase (MAP), which enhances viral infection [24]. Thus the resulting pathways are highly complex and designing rational strategies require intricate insight into the full complement of inhibitory and stimulatory kinase activities involved in its regulation and complete insight into the potential off-target effects of the drugs involved. Hence, there is a clear need for designing liver cell-compatible kinome profiling strategies.

## 3. Steatosis-Related Hepatitis

The abnormal retention of lipids in hepatocytes provokes hepatic steatosis, commonly referred to as fatty liver disease [25,26]. Risk factors associated with steatosis are varied but are usually related to obesity or alcohol abuse, which explains the apparently ever increasing incidence in steatosis-related liver disease [27]. The etiology of steatosis-related hepatitis is now fairly well understood [28] and prominently involves both endoplasmic reticulum (ER) stress [29] and activation of inflammasome signaling [30]. Cellular ER stress occurs upon the accumulation of misfolded proteins, but also as a result of accumulation of toxic lipids as is the case in steatosis. Misfolded proteins attract the ER sensor chaperone protein GRP78, which can bind and prevent aggregation of misfolded proteins. When this process fails, three separate unfolded protein response (UPR) mechanisms are activated, via the ER transmembrane molecules inositol-requiring kinase enzyme 1 (IRE1α), PRK-like ER kinase (PERK) and activating transcription factor 6 (ATF6) [31,32]. Activation of these pathways occurs either through the release of inhibitory GRP78 binding or direct activation through binding of unfolded proteins, and results in inhibition of protein translation and the activation of transcriptional patterns leading to increased protein folding machinery. In addition to two of the UPR sensors being kinases, their downstream targets also include other kinases, e.g., c-Jun N-terminal kinase (JNK) [33]. The UPR regulates lipid metabolism and through as yet unclear mechanism may contribute to steatosis [34]. Additionally, toxic lipids can directly activate the UPR sensors and promote steatosis in this way. ER stress signaling is complex and well known to be involved in a plethora of different gastrointestinal pathologies [35,36,37,38]. However it is fair to say that many of the mechanistic details are still unresolved, even if the principal involvement of different kinase activities is evident [39].

Inflammasome signaling is a common feature of inflammatory (infectious) diseases, where recognition of danger signals through a family of NOD-like receptors leads to assembly of large protein complexes (inflammasomes), the activation of which culminates in (amongst others) the production of (pro)inflammatory cytokines such as interleukin 1 (IL1β) and IL18. The best described inflammasome is NLRP3, which can be activated upon a wide variety of triggers [40] and activates Caspase 1 to promote its effects. In addition to alcohol, drugs and viruses, NLRP3/Caspase 1 may be activated through saturated fatty acids, and Caspase 1 knock out prevents hepatic steatosis in animal models [41,42]. In addition to the well described kinases SYK, JNK, IL-1 receptor-associate kinase (IRAK1), or Bruton’s tyrosine kinase (BTK), more recently it was suggested that Nima-related kinase-7 and TANK binding kinase (TBK1) play a role in activation of the NRLP3 core inflammasome [32]. Indeed, inhibition of SYK kinase activity was able to abrogate alcohol induced inflammasome signaling, resulting in reduced immune cell activation and hepatic steatosis in animal models, underscoring the importance of kinase modulation in the treatment of liver diseases [43].

Nevertheless, while a central role for kinase signaling is undisputed, much like ER stress signaling, much of the mechanistic detail of inflammasome signaling remains obscure at best, despite intense research effort [44]. Thus more insight into the biology of liver steatosis is needed and such knowledge may well yield novel targets for kinase-directed intervention of disease. Obviously unbiased kinome profiling approaches provide an attractive way forward here.

## 4. Liver Cancer

Oncological disease of the liver is especially prevalent in Asia and more recently in North Africa, but also in so-called Western countries liver cancer is a growing concern [45,46]. Liver cancer often presents as a result of ongoing inflammatory processes caused by hepatitis B or C, alcohol, or fatty liver disease. Despite evident promise, immune therapy for this disease remains unsatisfactory [47,48,49,50]. Importantly, the genetic basis of the disease is now partially understood [51,52,53] as is the resulting cell biology. Intrinsic factors such as stem cell markers (i.e., LGR5 [54]) and extrinsic factors including the surrounding microenvironment (e.g., bacterial and toxic drivers of mutagenesis [55] and cancer associated fibroblasts supporting cancer growth [56]) contribute to hepatocellular carcinoma growth. Importantly, aberrant cellular signaling, a result of DNA mutations of altered transcriptional profiles, is present in all cancers, including liver cancer. It has been described that the genetic alterations most commonly observed in HCC target 7 important signaling pathways in liver cancer: telomere maintenance, cell cycle regulation, Wnt/β-catenin signaling, oxidative stress pathways, epigenetic dysregulation pathways, AKT/mTOR signaling and Ras/Raf MAP kinase signaling [57]. The latter two are signaling pathways mainly driven by kinase activities. Copy number alterations in HCC frequently affect growth factors including VEGF and FGF [51]. The signaling cascades activated upon ligand binding to their tyrosine kinase receptors (VEGFR and FGFR) results in the activation of a plethora of kinases, including the above mentioned protein kinase B (PKB/AKT), mTOR, and the MAP/ERK (extracellular signal-regulated kinase) signaling route. Similar signaling pathways are activated by binding of MET to its hepatocyte growth factor receptor, which is also often amplified, overexpressed, or mutated in liver cancer [58,59]. Thus, targeting RTKs may be a promising strategy for HCC treatment [60]. Furthermore, amplification of the gene *PTK2* is also observed in up to 20% of HCC, resulting in overexpression of its encoded protein focal adhesion kinase (FAK), which is an important driver of tumor proliferation and metastasis [61]. It is generally recognized in the field that personalized approaches are the way forward [62], but they are hampered as inadequate of current NGS-panels for defining druggable mutations in liver cancer. Important work shows that targeting specifically multiple kinases hold promise in liver cancer [63] but the technological approaches involved do for now not allow introduction of such strategies in routine care [64]. It is speculated that kinome profiling approaches may allow efficient profiling of patient samples in cancer care [65]. Hence, if such approaches can be adapted as to allow routine assessment of clinical liver cancer specimens, treatment of this unusually lethal disease may be revolutionized.

While it is thus clear that diverse liver diseases would benefit from a better understanding of their kinomic changes, it should be noted that phosphorylation of proteins and lipids, is a resultant of two physiological processes: kinase activity and phosphatase activity. While kinases are able to transfer a phosphate group from an adenosine triphosphate (ATP) molecule to a specific substrate, hydrolysis of this phospho-esther bond is catalyzed by phosphatases, releasing inorganic phosphate. The resultant action of these two enzymes dictates cellular signaling. While kinases are abundantly studied, the role of phosphatases in disease states is less well investigated, as these enzymes are generally considered to be undruggable. With kinases representing oncogenes, the phosphatase is generally viewed as a tumor suppressor. This is certainly the case for some phosphatases, including the phosphatase and tensin homologue (PTEN) lipid phosphatase, which is frequently inactivated in tumors [66]. For instance, in HCC, PTEN expression levels are decreased, although this does not seem to be related to mutation of its gene [67]. Nevertheless, increasing evidence suggests that phosphatases may play a tumor promoting rather than suppressing function [68,69]. This has been linked to the release of inhibitory phosphorylation, as is the case for, for instance, the Src family of kinases [70,71,72]. The oncogene Src, the expression of which is enhanced in HCC, is kept in inactive formation through phosphorylation at its tyrosine residue 527 (Y527), while phosphorylation at Y416 causes its activation [73,74]. Thus, phosphatase enzymatic activity towards Y527 activates this oncogenic signaling pathway. Through these and other findings, phosphatases are now slowly being recognized as targets for cancer treatment, and even PTEN is now becoming a potential target for treatment [66,75,76].

While the human genome encodes around 500 kinase genes (of which 90 are tyrosine kinases [77]), only 107 phosphatases are recognized [78,79]. This suggests that kinase activity may be more tailored to individual cellular needs. As mentioned, kinases have been studied more apprehensively as compared to phosphatases. This has led to a larger arsenal of molecular tools to study kinases as compared to phosphatases, including antibodies and omics approaches. Thus, while phosphatases deserve more attention than they receive to date, analyzing phosphorylation in liver disease in bulk for now appears to be limited to kinome profiling, and therefore we will here focus on the tools to study this class of enzymes.

## 5. Need for Kinome Profiling Approaches in Liver Disease

Liver disease represents a tremendous burden on humanity and adequate therapeutic options are often lacking. It is well recognized that the liver physiology in general and disease in the liver in particular can be quite different as to what is encountered in other parts of the body (e.g., tissue resident memory T-cells in liver differs from those in other tissues [80]). Liver disease, in particular cancer, is very heterogeneous, and may vary by global region [81]. Generally speaking there is a paucity of technologies that can comprehensively characterize cellular physiology of the liver, especially those that are amenable for introduction into routine clinical practice. Kinome-wide study of hepatocyte and other liver cell kinase activity provides an attractive way forward here. Recent decades have seen an advent of technologies that potentially contribute to this (see, e.g., [82]). What are the possibilities in this respect for the study of the liver? Classically, researchers used in-gel kinase assays or in gel mobility shift of the protein induced by its phosphorylation, which could indirectly reflect the activity of a kinase, including liver cells [83]. With the identification of the importance of kinases in multiple diseases, the call for more efficient study technologies led to the conception of methods that were less laborious and yielded more accurate results. Especially the arrival of phospho-specific antibodies the late 1990s greatly improved the situation. However, there are hundreds of kinases that can be involved in different cellular activities and disease development. Studying these a masse would still present a huge workload, requires large volumes of samples to check all of the involved kinases by western blot and is dependent on antibody specificity. Therefore, researchers urgently need a rapid and large-scale method to measure overall cellular kinase activity. These days various kinome profiling methods provide answers here and can help untangling the complex role of kinases in liver disease.

### 5.1. MS-Based Kinome Profiling

MS-based kinome is based on mass spectrometry (MS) to detect phosphorylated proteins. While detection of total phosphoprotein content can be of great use, and results in large datasets informative of cellular phosphorylation status [84,85], we here describe two MS-based kinome profiling approaches, which are directed towards determination of activation of kinase activity.

**Figure 1:**
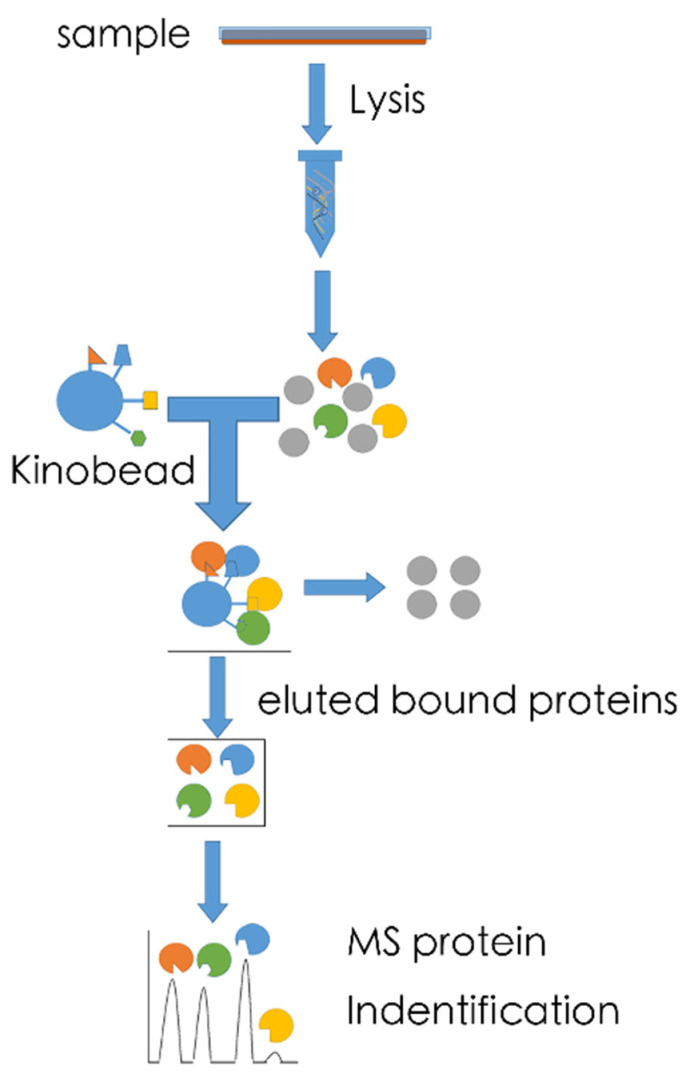
Kinobead-based profiling constitutes a chemical proteomics approach that entails the profiling of the interaction of small molecules with kinases. Following capture by immobilized kinase inhibitors the bound proteins are quantified in parallel by mass spectrometry using isobaric tags for relative and absolute quantification (iTRAQ) or comparable technology.

Kinobead-based profiling- The kinobeads assay has been reported by Bantscheff et al. in 2007 and can be considered a kinome profiling approach even if it does not directly measure kinase enzymatic activity as it allows identification of kinase inhibitors potentially active in the tissue from which the lysate was obtained (Figure 1) [86]. Its potential in liver disease was recently shown by the identification of kinase pathways that drive the epithelial–mesenchymal transition and drug resistance in hepatocellular carcinoma [87], a subject that regularly invites investigation through kinome profiling approaches (e.g., [88]). Broadly selective small molecule kinase inhibitors are immobilized on sepharose beads (Kinobeads). By binding in the ATP-binding pocket of kinases, affinity enrichment of native kinases from any tissue or cell lysate can be achieved. While such beads typically also catch other ATP-binding proteins, subsequent MS analysis can determine which peptides are derived from protein kinases. It should, however, be remembered that several factors, including overall levels of kinase expression and the affinity of the immobilized probes, affect kinase capture. For these reasons, confirmation of established activated kinases by secondary methods is important. Furthermore, this assay is primarily used to identify targets of kinase inhibitors and to quantify total expression levels of kinases rather than their activity. Nevertheless, this method has also been used to subsequently identify signaling events by phosphoproteome profiling of Kinobead-enriched samples [89]. Its advantages include the fact that it bypasses the drawbacks of antibody-based arrays, represents an unbiased approach to detect kinases, requires minimal volumes of sample and that the required inhibitor beads are standardized and can be manufactured in bulk. Accordingly, various companies, e.g., Cellzome use this technology, also for studying liver diseases, including parasitic ones [90], an endeavor for which it attracted in excess 33 million dollars from commercial parties. Such investments guarantee further development of this platform and it is thus to be expected that further successes, also for liver disease will follow.

**Figure 2:**
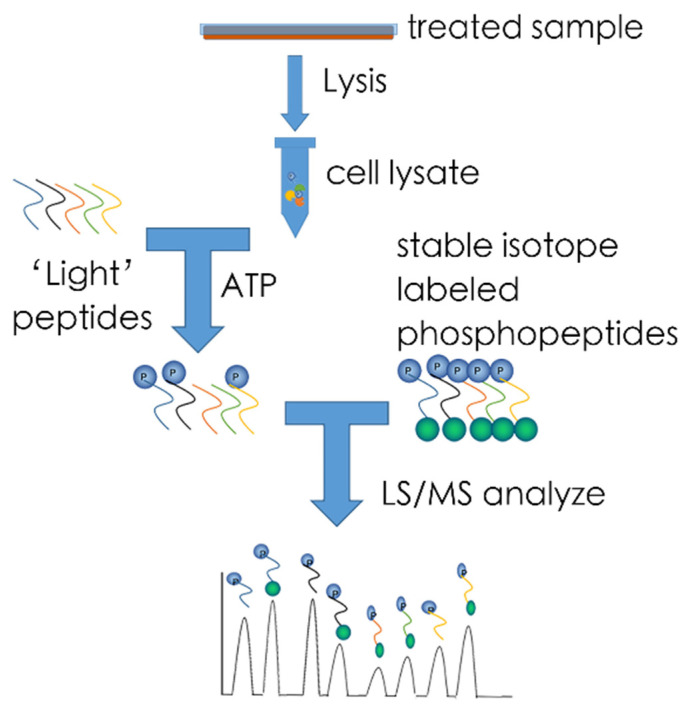
Kinase activity assay for kinome profiling (KAYAK). Synthetic substrate peptides are pooled and incubated with cell lysate. After quenching, isotope-labeled phosphopeptides of identical sequence to substrate peptides are added in a gnostic concentration to allow absolute quantification. Following enrichment of phosphorylated substrate peptides by immobilized metal-ion affinity chromatography results are obtained by analysis through LC–MS. Pairs of light and heavy peptides differ in mass and can be quantified by calculating the ratio.

The KAYAK approach—The KAYAK approach was first described by Yu et al. in 2009 [91] (Figure 2). The KAYAK (kinase activity assay for kinome profiling) method can measure up to 90 site-specific peptide phosphorylation events. It relies on the known substrate preferences of various protein kinases that are dictated by motifs surrounding the site of phosphorylation. For this method, 90 peptides with known kinase specificity are synthesized and added to cellular lysates in the presence of ATP. Phosphorylation of these target sequences can subsequently be identified and quantified by comparison with 90 stable isotope-labeled phosphopeptides, which are presynthesized as standard, and added to the sample at a known concentration. Both the sample’s peptide and the “heavy” peptide signal will be enriched by LS technique, after then captured by the MS technique and because of the size difference between the “light” peptide and the “heavy” peptide, the position and quantity of the target can easily be identified. While this method is relatively easy to deploy, the sensitivity of KAYAK approach is relatively low. Furthermore, many kinases show substrate promiscuity, and kinase specificity for their target sequences is not yet completely elucidated. Thus, the results of this method need to be interpreted with care. Nevertheless, its potential for sensitive multiplexed analysis of kinase activities and activity-based kinase identification makes it highly suitable for exploration hepatocyte or other liver cell type cellular physiology [92] especially when combined with highly precise MS technology like Orbitrap [93]. Exciting data

### 5.2. Array-Based Kinome Profiling

There are several strategies to detect the activity of kinases by array-based kinome profiling. Three array-based kinome profiling methods are detailed here.

**Figure 3:**
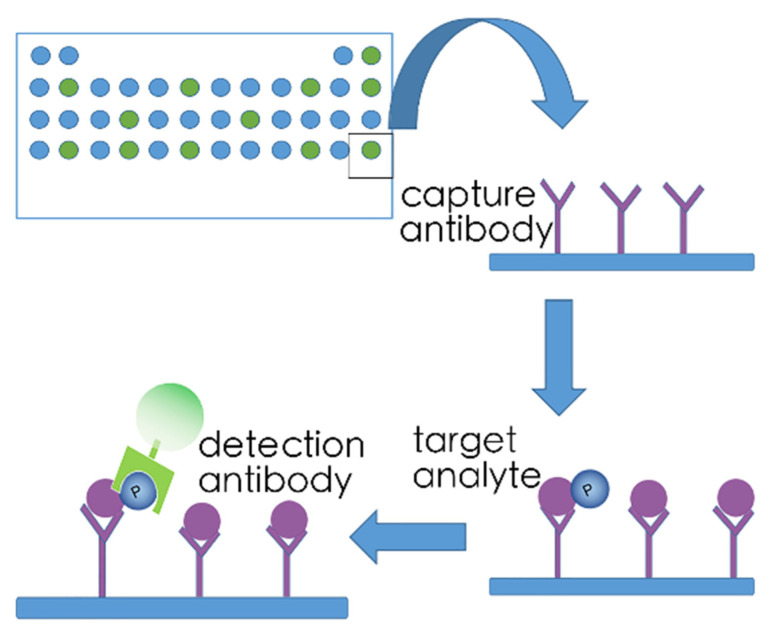
Phospho-kinase antibody array. Using specific capture antibodies spotted onto nitrocellulose membranes, the proteome profiler antibody array binds selected kinases when these membranes are incubated with experimental samples. A horseradish-peroxidase-coupled detection antibody, which recognizes phosphorylated tyrosine is added. Kinases are subsequently visualized by chemiluminescent detection reagents.

Phospho-kinase antibody array—One of the strategies to design kinome arrays is antibody-based. An example of this is the proteome profiler array, which can measure up to 119 proteins in a single sample (Figure 3). Using specific capture antibodies spotted onto nitrocellulose membranes, the proteome profiler antibody array binds selected receptor tyrosine kinases (RTKs) when these membranes are incubated with experimental samples [94,95]. A horseradish-peroxidase-coupled detection antibody, which recognizes phosphorylated tyrosine is added. Kinases are subsequently visualized by chemiluminescent detection reagents that generate a signal proportional to the amount of phospho-tyrosine antibody bound, allowing quantification of bound RTKs. No specialized equipment other than that used for conventional Western blotting techniques is required to use proteome profiler antibody arrays. This assay can easily be updated to fluorescent detection of bound antibodies. In addition, this assay is also available in 96 well formats, with detection of up to 16 receptor tyrosine kinases per well, allowing high throughput screening of larger number of samples. A drawback of this technique is that only receptor tyrosine kinases are detected, while the full cellular kinome includes many non-receptor kinases and serine/threonine kinases. However, arrays such as PathScan intracellular signaling array may provide outcome here, with detection of 18 serine, threonine or tyrosine phosphorylated intracellular targets, through direct binding of these phosphorylates forms with specific antibodies [95].

### 5.3. Kinase Target Sequence Arrays

**Figure 4:**
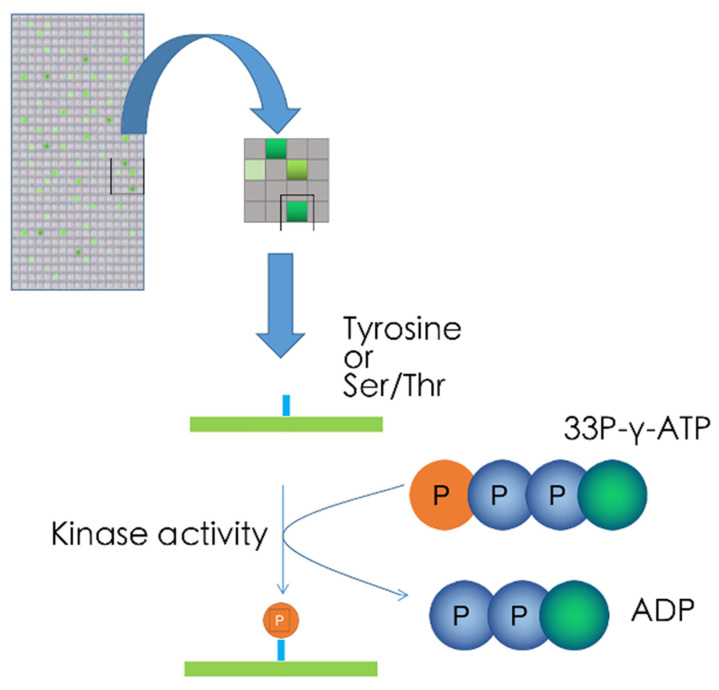
Peptide array-based profiling. Spatially addressed peptide substrates are immobilized and incubated with biological samples. Kinase activity is detected by incorporation of radioactivity into the substrates.

Target sequences of kinases (predicted in silico or derived from experimental evidence) can be synthesized onto glass arrays. Upon incubation of arrays with cellular lysates and 33P-*γ*-ATP, the terminal radiolabelled phosphor-group of ATP is transferred to a serine, threonine, or tyrosine residue when active kinases recognizing their target sequences are present in the cell lysate. Radiolabeled peptides can subsequently be detected by high-resolution phosphorimaging, allowing the simultaneous detection of hundreds of kinase reactions on one array (Figure 4) [96]. One commercially available example of such an array is Pepchip, produced in 2003 by the Pepscan System, which contains over 1000 target sequences and which remains in use for kinome profiling supporting high profile publications [97,98]. The advantage of this technique is the generation of a vast kinome network analysis from one single sample, and more importantly, the actual measurement of kinase activity in a sample rather than the phosphorylation patterns in a sample. Disadvantages include the use of radioactive materials, which may not be available in all research facilities, and, as mentioned above, the fact that kinase promiscuity towards peptide sequences may exists, complicating data analysis. In addition, phosphosites may contain more information than can be captured with peptide target sequences alone. For instance, some phosphorylation sits may only become phosphorylated when other phosphosite in the same protein are [99], which is something that cannot be detected using target sequences. Nevertheless, because of the relative large experience with this technology, which includes liver cells [100], it remains a highly promising platform for using kinome profiling for characterising liver pathophysiology.

### 5.4. Kinetic Kinase Target Sequence Arrays

**Figure 5:**
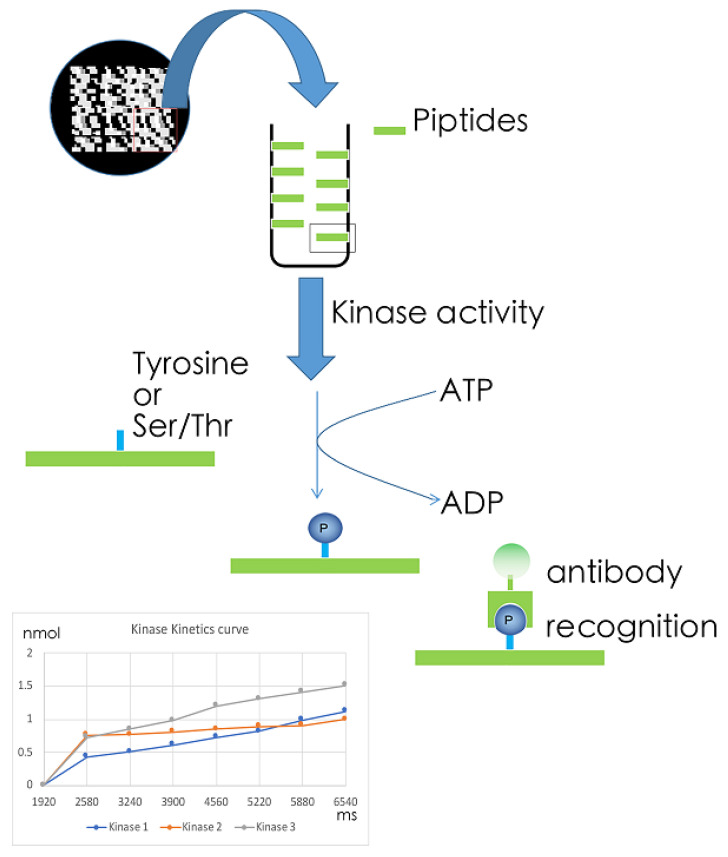
Kinetic kinase target sequence arrays. The microarray assay for kinase activity profiling is based on measuring peptide phosphorylation by protein kinases. The peptide sequences (13 amino acids long) harbor phosphorylation sites. Fluorescently labeled anti-phospho antibodies are used to detect phosphorylation activity of kinases present in the sample.

Instead of measuring phosphorylation of target sequences by radioactively labeled ATP, phospho-tyrosine or phosphor-serine/threonine specific antibodies can also be used [101,102]. A commercial example of this is PamChip (Pamgene), a chip containing four identical peptide arrays, each array containing 144 (STK) or 196 (PTK) peptide sequences of 13 amino acids long (Figure 5). Again, these peptides have been selected from the literature or computational prediction to be associated to one or several upstream kinases. During the kinase reaction, each peptide that has been phosphorylated will be marked by fluorescently labeled antibodies and images are taken every 5 min to generate real-time kinetics data [103,104]. Then, over time, complex outcomes can be generated. While other methods capture kinase activity or its outcome at one individual time point, this technique incorporates kinetic information, adding a layer of sensitivity. However, while current detection techniques allow simultaneous measurement of hundreds of kinetic reactions in one sample, static read out arrays (e.g., Pepchip) may contain up to one thousand kinase substrates. Nevertheless, because of low requirements with respect to input and very high reproducibility the kinetic technique is well suited for small diagnostic samples, e.g., liver biopsies.

## 6. Technical Noted on Measurements of Phosphorylation Levels

**Figure 6:**
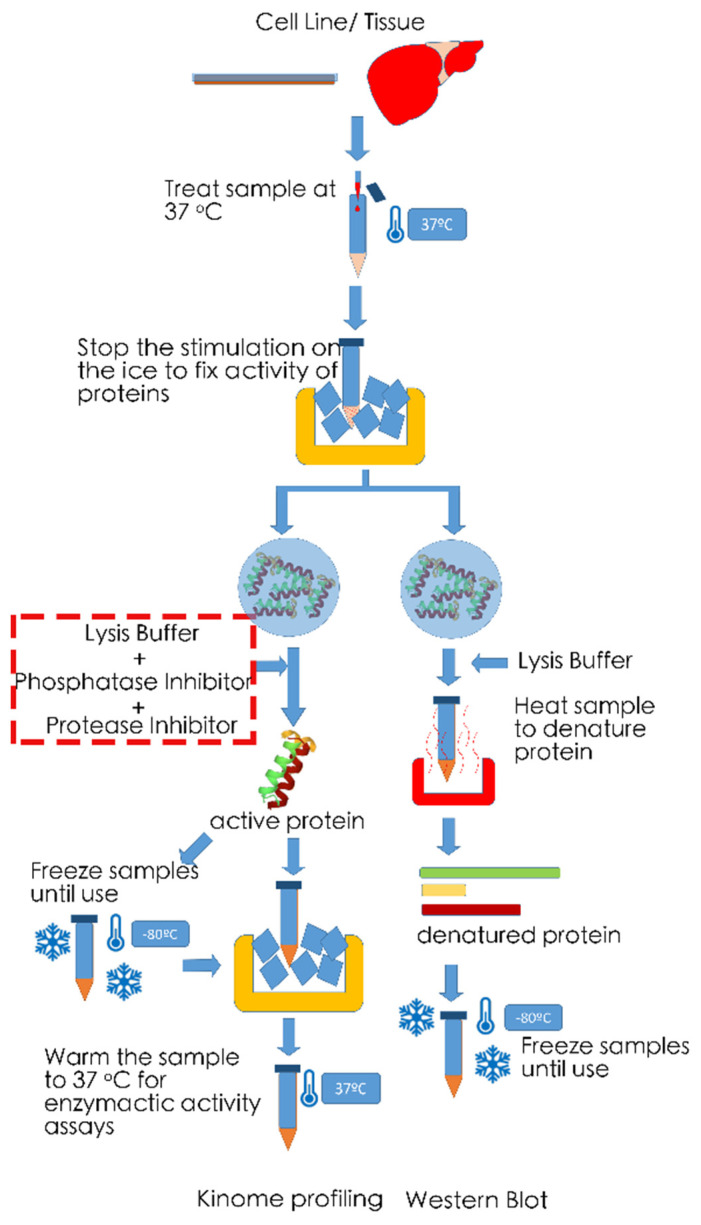
Analysis of native proteins for kinome profiling vs. denatured proteins for Western blot analysis. Kinome profiling can be performed on tissue samples or stimulated/unstimulated cells or cell lines. Placing cells on ice is important to fix the state of phosphorylation and enzymatic activity at the timepoint of interest. While for Western blot analysis samples are reduced and denatured by addition of (for instance) Laemmli buffer and boiling of the samples, proteins generally need to remain in their native state for kinome analysis. Mild lysis in the presence of protease inhibitors is recommended, and samples need to be kept cold at all preparatory steps. In order to reactivate resident enzymes, samples are heated to 37 °C for kinome applications depending on enzymatic activity.

An important note to make is one of a technical nature. Unlike Western blot analysis, kinome profiling approaches generally depend on the lysis of tissue samples and the extraction of their native kinases (see Figure 6). This is in order to allow capture of kinases in their native form by antibodies/chemicals and/or measurement of activity of the enzymes. As such, the obtained results will represent the overall kinase activity in all different cell types available in the sample, which in turn will largely depend on the quality of the tissue. For instance, one should ensure that tissues are immediately snap-frozen or immediately lysed upon collection of specimens to guarantee that differences in degradation of the proteins in different samples do not influence results. Addition of phosphatase and protease inhibitors is recommended in this respect. Additionally, when comparing stimulated vs. unstimulated samples (e.g., for primary cells or cell line experiments), it is essential to terminate reactions on ice to prevent kinases and phosphatases to further affect cellular phosphorylation levels and thus kinase activities. Furthermore, protein quantification prior to analysis is imperative to ensure that differences in kinase activities observed are not the result of loading different protein quantities. Unlike Western blot analysis, the use of a housekeeping protein is not yet commonplace for kinome profiling assays.

It should also be noted that when using tissues, the composition of the tissue is of importance. For instance, fibrosis may hamper protein extraction, differences in tumor infiltration of immune cells may confound results, and in our experience array-based kinome approaches are very sensitive to sample lipid content (unpublished data), which may make profiling steatotic liver tissue using these techniques more challenging. Upon lysis of samples, spatial information of the tissues is no longer available and it is impossible to attribute kinase activities to any given cell type in a tissue. Mass imaging efforts for special resolution (e.g., Cytometry by Time-Of-Flight (CYTOF) mass spectrometry) are up and coming [105], but do not allow assessment of kinase activities, nor are they as yet specifically directed towards determination of phosphorylation events. All in all, careful consideration of the tissues used to determine global kinome profiles is of the essence and should be the basis of all experimental set-ups.

## 7. Future Perspectives on the Application of Kinome Profiling in Liver Disease

**Figure 7:**
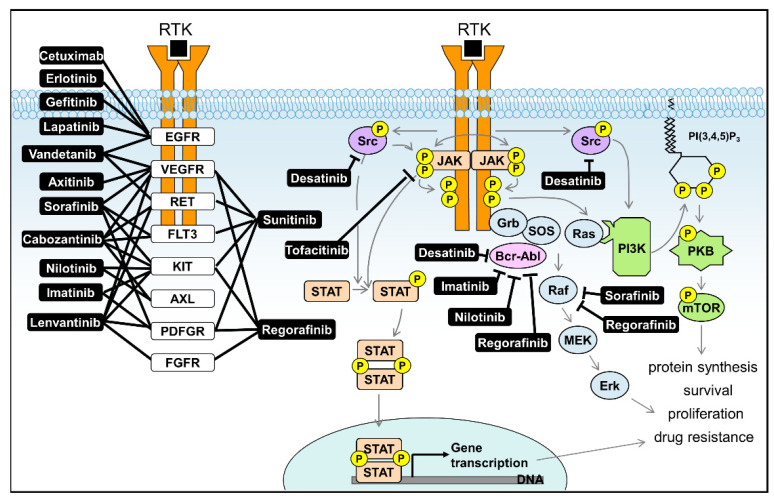
Common signaling pathways in liver disease (primarily HCC) and the kinase inhibitors aimed at these pathways. Receptor tyrosine kinase (RTK) inhibitors are the main group of kinase inhibitors currently used in clinical practice. Most RTKs activate common downstream pathways: the JAK/STAT pathway, the mitogenic Ras/Raf/MEK/ERK pathway, and the PI3K/PKB survival pathway. These pathways show considerable overlap, with Src activating several of their members, and the guanine nucleotide exchange factor SOS being a target for both the adaptor protein Grb (activated via RTKs) and the oncogene Bcr-Abl (Figure based on [69,123]).

The advent technologies capable of comprehensively describing the cellular kinome now opens the door for improved understanding of liver diseases and the possibilities for designing novel avenues for the rational treatment of hepatic disease. There is substantial support to be found in the literature that such endeavors may prove successful as it is becoming more and more clear that many diseases located in the liver involve activity of specific kinases and might thus be targeted by specific inhibitors even if the identity of the kinases involved remains unclear (Table 1). Currently, despite the fact that most cellular phospho-content is represented by serine/threonine phosphorylation, scientific focus is mainly drawn to the use of tyrosine kinase inhibitors, in particular those inhibiting receptor tyrosine kinases and their downstream targets (Figure 7). Serine/threonine kinase inhibitors, while gaining interest [106], are as yet underrepresented in the clinic [107]. For liver diseases, the notable exceptions are for instance Raf inhibitors such as Sorafinib and Regorafinib. This leaves huge scope for finding alternative treatments. Obviously kinome profiling is well-suited to address this void and conjunction with recent technical advances, it is to be expected that investigators will grab the opportunity at hand.

**Table 1:** Common inhibitors of kinases and their target.

Inhibitor	Target	Cellular Signalling	Type	FDA Approved	Studied in Liver Disease
Axitinib	VEGFR	Receptor tyrosine kinases inhibitor	Small Molecule	Approved for RCC	Phase II completed for HCC [108]
Cetuximab	EGFR	Receptor tyrosine kinases inhibitor	Monoclonal antibody	Approved for CRC	Phase II completed for HCC [109]
Cabozantinib	VEGFR/KIT/TRKB/FLT-3/AXL/RET/MET/TIE-2	tyrosine kinase inhibitor	Small Molecule	Approved for MTC	Phase III Active Not Recruiting for HCC [110]
Dasatinib	BCR-ABL/Src kinase family	tyrosine kinase inhibitor	Small Molecule	Approved for CML	Phase II Terminated for HCC [111]
Erlotinib	EGFR tyrosine kinase	Receptor tyrosine kinases inhibitor	Small Molecule	Approved for NSCLC	Phase III completed for HCC [112,113]
Gefitinib	EGFR tyrosine kinase	Receptor tyrosine kinases inhibitor	Small Molecule	Approved for NSCLC	Phase II completed for HCC [114]
Imatinib	Bcr-Abl/KIT/PDGFR	tyrosine kinase inhibitor	Small Molecule	Approved for CML	Preclinical models for liver fibrosis [115]
Lapatinib	EGFR/HER2	Receptor tyrosine kinases inhibitor	Small Molecule	Approved for Breast Cancer	Phase II completed for HCC [116]
Lenvatinib	VEGFR/FGFR/PDGFRα/KIT/RET	Receptor tyrosine kinases inhibitor	Small Molecule	Approved for HCC	Phase III enrolling for HCC [117]
Nilotinib	BCR-ABL	tyrosine kinase inhibitor	Small Molecule	Approved for CML	Preclinical models for liver fibrosis [118]
Sorafenib	CRAF/BRAF/KIT/FLT-3/VEGFR-2/VEGFR-3/PDGFR-ß	Raf kinase/Receptor tyrosine kinase inhibitor	Small Molecule	Approved for HCC	Phase III enrolling for HCC [119,120]
Sunitinib	PDGFRa/PDGFRb/VEGFR1/VEGFR2/VEGFR3/KIT/FLT3/CSF-1R/RET	Receptor tyrosine kinase inhibitor	Small Molecule	Approved for RCC and GIST	Phase III Terminated for HCC [119]
Vandetanib	VEGFR/EGFR/RET	Receptor tyrosine kinase inhibitor	Small Molecule	Approved for MTC	Phase II completed for HCC [121]
Regorafenib	RET/VEGFR1/VEGFR2/VEGFR3/KIT/PDGFR-alpha/PDGFR-beta/FGFR1/FGFR2/TIE2/DDR2/TrkA/Eph2A/RAF-1/BRAF/BRAFV600E /SAPK2/PTK5/BCR-ABL	Raf kinase/tyrosine kinase inhibitor	Small Molecule	Approved for CRC, GIST and HCC	Phase III completed for HCC [120]
Tofacitinib	JAK1/JAK2/JAK3/TYK2	Janus kinases inhibitor	Small Molecule	Approved for severe rheumatoid arthritis	Phase I completed for Hepatic Insufficiency [122]

Abbreviations: RCC, Renal cell carcinoma; HCC, hepatocellular carcinoma; CRC, Colorectal cancer; MTC, Medullary thyroid cancer; CML, Chronic myelogenous leukemia; NSCLC, Non-small-cell lung carcinoma.

### 7.1. Alcoholic Liver Disease

Alcohol abuse and especially alcoholism remains a primary cause of liver dysfunction, especially because of the development of portal hypertension, which results from hepatocyte steatosis, subsequent cell death and liver fibrosis, and the alcoholic hepatitis per se [124]. Currently, pharmacological options to treat alcoholic liver disease are very limited and are confined to therapy supporting abstinence of alcohol (e.g., stimulators of GABA-ergic transmission [125]), therapies aimed at mitigating the effects of portal hypertension (e.g., [126]) or supporting liver function [127]. The paucity of options in this respect is also driven by a relatively surprising extent of insight into the molecular mechanisms that drive disease. Intriguingly, however, the evidence points to an important role of kinase enzymes in disease progression. A recent multiomics attempt to improve insight into alcoholic liver disease, involving the analysis of liver tissue from patients with alcoholic hepatitis and alcoholic cirrhosis and its contrast to normal liver tissue obtained from hepatic resection, identified hexokinase domain containing 1 kinase as the most distinctive characteristic of alcoholic disease (although kinase activity itself was not assessed in this study) and high levels were also associated with poor survival [128]. Analogously, increased activity of the NF-kB signal transduction-regulating kinase IRAK4 phosphorylation were found in livers of patients with alcoholic hepatitis [129], suggesting that strategies aimed at combating inflammatory signaling in alcoholic hepatitis could be specifically targeted at this kinase. In addition, it is understood that fibroblast growth factor (FGF) 21 is important in the progression of alcoholic cirrhosis [130], but which of the six FGF receptors potentially involved is mediating these effects is unknown and kinome profiling may help its identification. A further lead may be the expression of Tec kinase that appears specifically associated with the progression of alcoholic hepatitis [131]. The notion that a specific hepatic alcohol-related disease kinase department exists is bolstered by the observation that NASH but not alcoholic liver disease is associated with SYK activity [132,133]. It is thus rational to assume that specific kinases can be identified in alcoholic liver disease whose inhibition should prove clinically exceedingly useful. Kinome profiling platforms form the rational choice for identifying these kinases.

### 7.2. Liver Cancer

Sorafinib is currently almost uniquely a therapeutic option in the treatment of HCC [134], providing modest increase in the life expectancy of the patients involved. Although the relevant target has not yet been conclusively identified [5], it is thus evident that HCC may be a target for specific therapy with kinase inhibitors. Kinome profiling may provide a way the relevant kinase target and especially in vitro experiments on liver cancer organoids (which also react to sorafibib therapy [54,56]) in combination with comprehensive kinome profiling may well unequivocally answer questions in this respect. Generally speaking, many kinases have emerged as potential leads in the development of novel therapy for HCC, including AMPK [135] and a variety of receptor tyrosine kinases [136], e.g., the Axl receptor tyrosine kinase attracts attention in this respect [137]. While kinome profiling approaches in general can support studies aimed at describing enzymatic activities in kinase compartment, using temporally sequential biopsies during experimental treatment of patients with inhibitors should greatly facilitate understanding the promise of experimental medicine and drive development of improved strategies. Especially the Pamgene platform is a promising candidate here, as it requires very little input material and is exquisitely sensitive to small changes in the enzymatic characteristics of receptor tyrosine kinases. Indeed, employing mass spec kinome approaches have already begun to be pursued in HCC, see, e.g., the recent studies by Ren et al. [138] or the RNAi library approach [139] by Chen et al. [140]. Hence, the look out for kinome profiling for research into improved treatment of HCC is very favorable.

Apart from HCC the other main modality of liver cancer is cholangiocarcinoma. This disease is unusually treatment resistant and apart from surgical resection, no curative options exist, while anticancer palliative options are also lacking [141]. For now, targeted options with respect to kinase inhibition for the treatment of cholangiocarcinoma have proven disappointing [142,143]. Nevertheless, the kinase compartment does appear instrumental in driving the disease process, e.g., the fairly aspecific kinase inhibitor dinaciclib suppresses cholangio carcinoma xenograft growth, possibly by targeting cyclin dependent kinases [144]. The elucidation of activity of kinase compartment in this disease and especially the relation of the activities detected to eventual patient survival, through kinome profiling may shed important light and might at least suggest possible novel direction for research into improved treatment.

### 7.3. Hepatitis E

Hepatitis E is a global health issue [11] and currently no FDA-approved drugs are available for its treatment, even if off-label treatment with the side effect-prone ribavirin is common [145]. Library approaches to identify drugs potentially inhibiting HEV replication have proven successful and tend to lead to the identification of modulator of kinase activity [146]. Further progress will await, however, better understanding of the exact changes in kinase activity in the liver cells of the patients affected by the disease. HEV biology remains relatively uncharacterized in this respect [147]. The availability of kinome platforms and the presence of research groups interested both in kinome profiling and in HEV biology should foster quick implementation of kinome profiling technology for better understanding of HEV-provoked kinase signaling and the importance of the kinases identified for the biology of the virus [18,148].

### 7.4. Wilson’s Disease

Wilson’s disease, which is a copper dyshomeostasis, is an autosomal recessive disease that presents mainly with hepatic, neurological, and psychiatric manifestations [149]. The disease results from the absent or reduced function of the ATP7B transporter that is important for biliary excretion of copper and the incorporation of copper in caeruloplasmin [150]. The only potentially curative treatment is liver transplantation, but in view of the paucity of suitable donor livers and competition with other disease modalities, patients can remain on the waiting list for orthotopic transplantation for prolonged times. Thus pharmacological management options for this disease are urgently called for. Intriguingly, a recent high profile study identified AMPK activity in a mouse model of this disease as significantly increased, while also the insulin receptor tyrosine kinase emerged as a possible mediator of pathophysiology [151]. Other preclinical studies pointed to the involvement of p38MAP kinase in the pathogenesis of Wilson’s disease [152]. Thus the evidence that kinase signaling constitutes a viable and bona fide target is quite strong, but good data obtained in clinical samples are lacking, The power of kinome profiling using a peptide array to generate comprehensive descriptions of cellular kinase activity in combination with the availability of explant livers to support experimentation on human patient material provides an obvious opportunity here, which will certainly be taken by groups active in translational research in this disease.

## 8. Conclusions

There is an urgent need for studies generating comprehensive descriptions of cellular kinase activities in liver diseases and because competing technologies for now fail to deliver diagnostic possibilities for routine care, there is opportunity for kinome profiling in this respect. Various technologies capable of filling this void are available. The coming years will reveal whether investigators and companies active in this field will grab the opportunity available.
